# Serotonergic innervation of the amygdala is increased in autism spectrum disorder and decreased in Williams syndrome

**DOI:** 10.1186/s13229-019-0302-4

**Published:** 2020-02-05

**Authors:** C. H. Lew, K. M. Groeniger, K. L. Hanson, D. Cuevas, D. M. Z. Greiner, B. Hrvoj-Mihic, U. Bellugi, C. M. Schumann, K. Semendeferi

**Affiliations:** 1 Department of Anthropology, University of California, San Diego, USA; 2 Department of Biological Sciences, University of California, San Diego, USA; 3 Salk Institute for Biological Sciences, San Diego, USA; 4 Department of Psychiatry and Behavioral Sciences, University of California, Davis School of Medicine, the MIND Institute, Sacramento, USA

## Abstract

**Background:**

Williams syndrome (WS) and autism spectrum disorder (ASD) are neurodevelopmental disorders that demonstrate overlapping genetic associations, dichotomous sociobehavioral phenotypes, and dichotomous pathological differences in neuronal distribution in key social brain areas, including the prefrontal cortex and the amygdala. The serotonergic system is critical to many processes underlying neurodevelopment and is additionally an important neuromodulator associated with behavioral variation. The amygdala is heavily innervated by serotonergic projections, suggesting that the serotonergic system is a significant mediator of neuronal activity. Disruptions to the serotonergic system, and atypical structure and function of the amygdala, are implicated in both WS and ASD.

**Methods:**

We quantified the serotonergic axon density in the four major subdivisions of the amygdala in the postmortem brains of individuals diagnosed with ASD and WS and neurotypical (NT) brains.

**Results:**

We found opposing directions of change in serotonergic innervation in the two disorders, with ASD displaying an increase in serotonergic axons compared to NT and WS displaying a decrease. Significant differences (*p* < 0.05) were observed between WS and ASD data sets across multiple amygdala nuclei.

**Limitations:**

This study is limited by the availability of human postmortem tissue. Small sample size is an unavoidable limitation of most postmortem human brain research and particularly postmortem research in rare disorders.

**Conclusions:**

Differential alterations to serotonergic innervation of the amygdala may contribute to differences in sociobehavioral phenotype in WS and ASD. These findings will inform future work identifying targets for future therapeutics in these and other disorders characterized by atypical social behavior.

## Background

Williams syndrome (WS) is a rare neurodevelopmental disorder (~ 1/10,000 [1]) caused by a hemizygous deletion on chromosome band 7q11.23 and associated with a distinct socioaffective phenotype which includes an atypically strong drive for social engagement, an uninhibited propensity to approach and socially engage with strangers, decreased social anxiety, and increased attention to faces [2, 3]. In contrast, autism spectrum disorder (ASD) is a common neurodevelopmental disorder (1/59 in the USA [4]) with a highly complex and heterogeneous genetic etiology and a behavioral phenotype characterized in part by reduced drive for social engagement and decreased attention/atypical processing of the eyes of others, an important social stimulus in humans [5, 6]. Our previous studies in the postmortem brains of individuals with ASD [7] and WS [8, 9] have demonstrated opposing patterns of difference compared to healthy controls in the number of neurons in the same key social brain areas, paralleling differences in social behavior. Together, these findings suggest that a direct comparison of these two disorders may offer a unique human model in which to examine changes in the brain that may contribute to the biological underpinnings of social behavior and, furthermore, may help elucidate critical neural targets for potential therapeutics in disorders accompanied by sociobehavioral difficulties.

The amygdala, a limbic structure located in the medial temporal lobe, is critically implicated in social behavior and emotion. Neuroimaging studies have demonstrated structural and functional abnormalities of the amygdala in many neurological disorders that are accompanied by atypical social behavior, including in the WS and ASD amygdala [10–13]. However, the relationship between structure and function of the amygdala and behavior remains elusive. The amygdala is composed of several nuclei that can be distinguished from each other based on histological criteria [14], and tracer and lesion studies in animal models suggest that the structural heterogeneity of these nuclei correspond to functional differences. Four nuclei in particular, the lateral, basal, accessory basal, and central nuclei, are significantly implicated in two distinct but overlapping processing loops. The lateral, basal, and accessory basal nuclei are thought be involved in cognitive processing given the significant bidirectional connectivity to association areas in the frontal and temporal lobes [15–17]. In contrast, the central nucleus is critical to the autonomic loop of processing in the amygdala, as it lacks connectivity to association cortex, but receives heavy intra-amygdala projections, and serves as the major output nucleus of the amygdala to brain stem and hypothalamic regulatory centers [15–17]. In our postmortem studies of the amygdala in WS [9] and ASD [7, 18], we found that the lateral nucleus was selectively vulnerable in both disorders, such that compared to NT, there was a significant increase in neuron number in the lateral nucleus in WS and a significant decrease in neuron number in the lateral nucleus in ASD. The lateral nucleus is the primary site of cortical input into the amygdala and an important region for cognitive processing of external stimuli, so these targeted alterations, in opposing directions of change, may contribute to differential atypical processing of social stimuli in WS and ASD.

While differences in neuron number likely contribute to differences in amygdala function, neuronal activity is frequently modulated by neurotransmitter systems. Serotonin is a monoamine that has been implicated in a diverse array of functions in the brain. As a neurotransmitter, serotonin plays a role in several processes of neural development and neural plasticity, including neurogenesis, neural differentiation, axon myelination, and synapse formation and remodeling [19, 20]. Serotonin is also a key neuromodulator in several processes of emotion and cognition, including anxiety and social behavior [21]. WS and ASD diagnoses share a high comorbidity with anxiety disorders, and the effective use of selective serotonin uptake inhibitors (SSRIs) in mitigating symptoms of severe anxiety in patients with WS and ASD implicates involvement of the serotonergic system in both disorders [22, 23]. Furthermore, studies in animal models have found evidence of altered serotonergic metabolism and synthesis in WS and ASD that are associated with characteristic behavioral and neuroanatomical phenotypes [24–26]. Neuronal activity in the amygdala is heavily modulated by serotonergic axons, and disruptions to amygdala serotonergic chemoarchitecture may contribute to neuropathologies underlying atypical social behavior, such as the dichotomous behavioral phenotypes of WS and ASD.

A key component of the serotonergic function within the brain is serotonin transporter (SERT), which is involved in serotonin reuptake back into the presynaptic terminal. Maternal SERT function has been demonstrated to have a profound effect on neural development in offspring in animal models [27]. Animal studies have additionally found significant associations between SERT expression and behavior [28, 29]. In humans, histological methods that label SERT expression in preserved brain tissue can give insight into the chemoarchitecture and anatomy of the serotonin system. Atypical SERT axon density in postmortem brains has been observed in the cortex in other neurological disorders with affective behavioral phenotypes, including schizophrenia [30] and victims of suicide [31, 32]. However, no study to date has quantified SERT axon density across the major subdivisions of the postmortem human amygdala in any disorder or disease, including ASD and WS. Here, we utilized immunohistochemical methods to determine the density of SERT immunoreactive (SERT-ir) axons in the lateral, basal, accessory basal, and central nuclei of the amygdala in WS and ASD, and we compared these results with our data on SERT-ir axon density in neurotypical (NT) postmortem brains, as previously reported in Lew et al. [33], in order to test the hypothesis that serotonergic chemoarchitecture of targeted amygdaloid nuclei are disrupted in ASD and WS. Specifically, given previous qualitative observations of global increases in SERT axon density in ASD [34, 35] and a pattern of opposing directions of change in WS and ASD cytoarchitecture [7, 8, 18], we predicted SERT axon density of the amygdala would be increased in ASD and decreased in WS compared to NT and that the basolateral nuclei would demonstrate the greatest differences between the two disorders.

## Methods

The data sets included in this study were obtained from the postmortem amygdala of a total of 20 subjects, composed of six age-matched adult sets (NT, ASD, WS) and one age-, sex-, and hemisphere-matched WS-NT infant pair (see Table 1 for subject background). A corresponding ASD infant could not be included in this study, as ASD is not formally diagnosed until around 2.5 years of age at the earliest [36]. The data set obtained from the six adult NT subjects was previously reported by us in an earlier publication [33]. The adult WS and ASD tissue and the WS-NT infant pair was processed and data was collected following identical methods. Only subjects free of seizures or other neurological disorders were used. Amygdala tissue from individuals diagnosed with ASD prior to death was obtained from the laboratory of Cynthia Schumann (MIND Institute, UC Davis School of Medicine). Amygdala tissue from individuals diagnosed with WS derived from the Ursula Bellugi Williams Syndrome Brain Collection, an ongoing donation-based program run by the Laboratory for Human Comparative Neuroanatomy at UC San Diego (Semendeferi, PI), in collaboration with the NIH NeuroBioBank at the University of Maryland. Fluorescence in situ hybridization (FISH) probes for elastin, a gene consistently deleted in the WS hemideletion, were used to determine genetic diagnosis in the WS cases, and all WS subjects used in this study demonstrated the typical WS genetic deletion. Mutations and deletions of the elastin gene are associated with supravalvular aortic stenosis, a heart defect that is prevalent in WS, and notably, cardiac complications was the cause of death in five of the seven WS subjects included in this study. Diagnosis for ASD subjects was assessed based on results of the Autism Diagnostic Interview-Revised and other medical records [7]. Mean age and age range for adult subjects were similar across all three groups included in the analysis (age mean, age range in years: NT = 45, 19–69; ASD = 41, 20–64; WS = 41, 17–69). One hemisphere (right or left, based on availability) was analyzed per subject. A lack of hemispheric asymmetry has been observed in the human amygdala in both histological and neuroimaging studies [37, 38] suggesting a single hemisphere is sufficient for analysis. Diagnostic groups were not matched for sex or hemisphere due to the limited availability of postmortem human brain tissue, and particularly brain tissue from individuals with neurodevelopmental disorders, which is exceptionally rare.

**Table 1:** Subject background

Subject ID	Age at death	Diagnosis	Sex	Hemisphere	Cause of death	Postmortem interval (hours)
5183	107 days	Neurotypical	Male	Right	Sudden infant death syndrome	13
WS 7	114 days	Williams syndrome	Male	Right	Multiple organ failure	30
WS 10	17 years	Williams syndrome	Male	Right	Cardiac complications	24
4916	19 years	Neurotypical	Male	Right	Drowning	5
H-10-01	20 years	Autism spectrum disorder	Male	Right	Motor vehicle accident	24
WS 15	25 years	Williams syndrome	Female	Right	Cardiac complications	30
H-11-02	27 years	Neurotypical	Male	Left	Cardiovascular disease	16
H-6-04	28 years	Autism spectrum disorder	Male	Left	Monoxide poisoning	18
WS 14	42 years	Williams syndrome	Female	Right	Cardiac complications	18
H-7-02	42 years	Autism spectrum disorder	Male	Right	Cardiac arrest	24
WS 9	43 years	Williams syndrome	Female	Right	Cardiac complications	12
5758	43 years	Neurotypical	Female	Right	Sepsis	22
H-6-00	44 years	Autism spectrum disorder	Male	Left	Cardiovascular disease	31
H-19-01	44 years	Neurotypical	Male	Left	Unknown	26
H-1-01	44 years	Autism spectrum disorder	Male	Left	Pulmonary Embolism	20
WS 8	48 years	Williams syndrome	Male	Left	Respiratory illness	30
H-4-02	64 years	Autism spectrum disorder	Male	Right	Sepsis	18.5
H-11-98	67 years	Neurotypical	Male	Left	Unknown	?
WS 13	69 years	Williams syndrome	Male	Right	Cardiac complications	8
5943	69 years	Neurotypical	Male	Right	Acute coronary artery thrombosis	23

### Tissue processing

One brain hemisphere from each subject was immersed in 10% buffered formalin after autopsy (see Table 1 for postmortem interval) and remained in formalin until sectioning. Tissue blocks containing the entire rostrocaudal extent of the amygdala were extracted from the whole hemisphere of the brain. Extracted blocks were saturated in a cryoprotectant solution of sucrose and 0.1 M phosphate buffer, frozen with dry ice, and cut along the coronal plane using a Leica SM sliding microtome. Tissue was cut in either alternating 80 μm and 40 μm sections (WS tissue and NT tissue from NIH NeuroBioBank) or alternating 100 μm and 50 μm sections (ASD tissue and NT tissue from the Schumann collection). A 1-in-10 series of either 80 μm or 100 μm sections per individual was mounted and stained for Nissl substance, and a 1-in-20 series of either 40 μm or 50 μm sections per individual was stained with mouse monoclonal antibody against SERT (MAB5618, EMD Millipore, Billerica, MA) using the heat-based antigen retrieval and immunohistochemical staining protocol described in our previous publication [33].

### Data collection

**Fig. 1:**
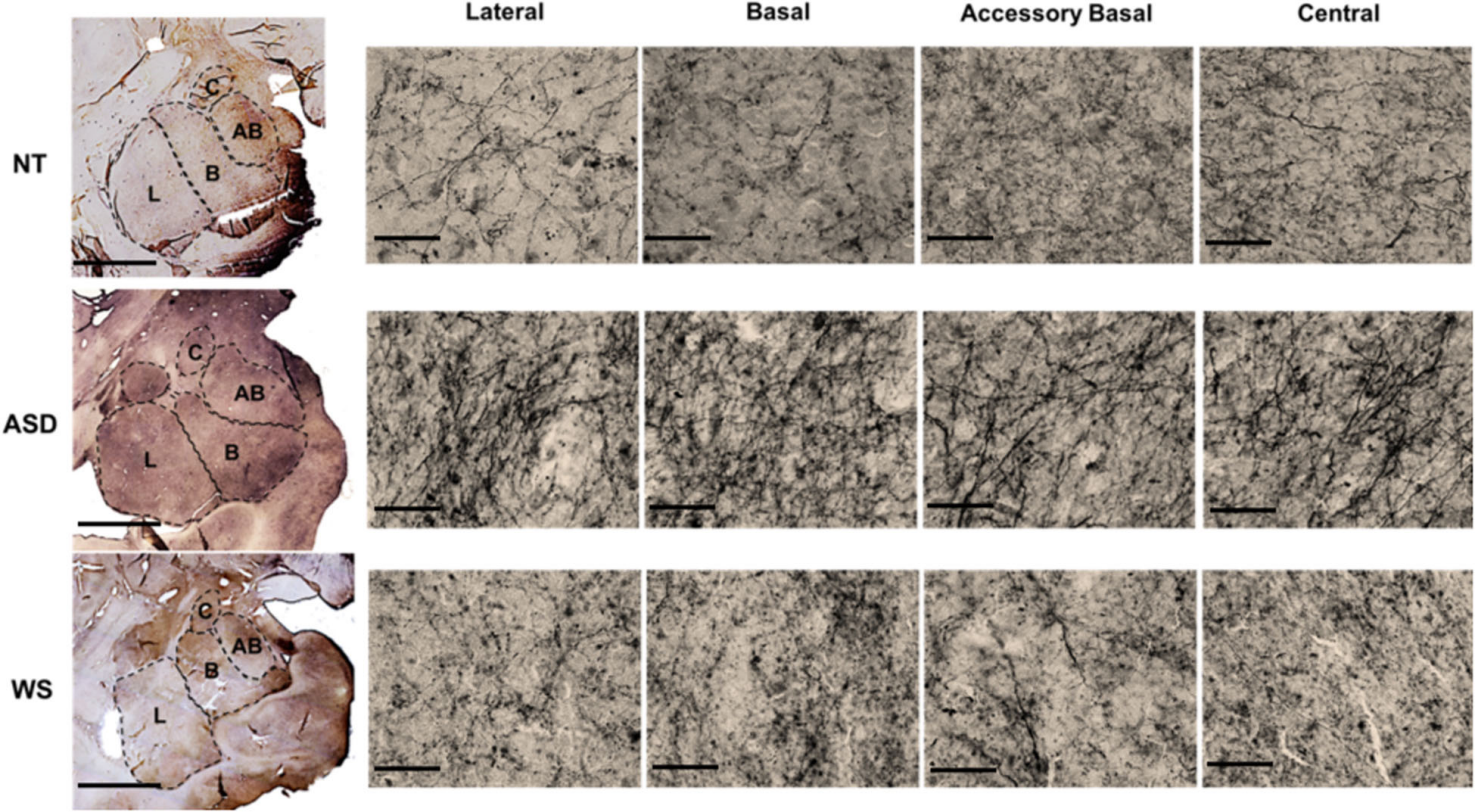
Micrograph showing the four regions of interest in the amygdala in each diagnostic group. The first photo of each row shows the whole amygdala with boundaries of the lateral, basal, accessory basal, and central nuclei (scale bar = 5 mm). The remaining photos in each row show SERT-ir stained fibers in each nucleus examined at × 60 magnification (scale bar = 50 μm)

Adult data was collected by CL and infant data was collected by KG, after establishing inter-rater reliability with > 95% concordance. Data was collected using the Stereoinvestigator software suite (MBF BioScience, Williston, VT) on a Dell workstation with a 30.48 centimeter (cm) by 53.34 cm monitor, receiving live video feed from a Lumenera color video camera (Ottawa, Ontario) attached to an Eclipse 80i microscope equipped with a Ludl MAC5000 stage (Hawthorn, NY) and a Heiden z-axis encoder (Plymouth, MN). For each section examined, boundaries of the amygdaloid nuclei were first traced in Stereoinvestigator at × 1 magnification, utilizing an adjacent section from the Nissl-stained series as a visual aid during tracing to ensure precision of boundaries (described in detail in [9, 14]). After boundaries on the SERT-ir stained sections were identified (Fig. 1), the Stereoinvestigator Spaceballs probe, which utilizes systematic random sampling for accurate stereologic quantification, was employed to estimate SERT-ir axon length at × 100 magnification (1.4 numerical aperture, oil lens), using the parameters described in our previous publication [33]. Total axon length density was calculated by dividing the total axon length by the planimetric reference volume [39, 40].

### Analyses of data

All data analyses were performed using Prism statistical software (v.8, GraphPad Software, La Jolla, CA). Spearman rank-order correlation tests were used to identify any age, sex, or post-mortem interval (PMI) effects on SERT-ir axon density, and data for all subjects was run through a Grubbs’ test (*P* < 0.05) to detect possible outliers. Given the small sample size of the data sets, non-parametric statistical methods were used. The Kruskal-Wallis test with Dunn’s test for multiple comparisons was employed to examine differences in SERT-ir axon density in the lateral, basal, accessory basal, and central nuclei between each group. While the infant pair was included in the analyses of NT-WS comparisons, only adult subjects were included in NT-ASD and ASD-WS comparisons due to the unavailability of an ASD age-matched infant. The difference between mean density of SERT-ir axons in ASD and WS was calculated as the percentage of mean density in NT subjects (only adult NT subjects included for comparison with ASD; all NT subjects included for comparison with WS).

## Results

**Fig. 2:**
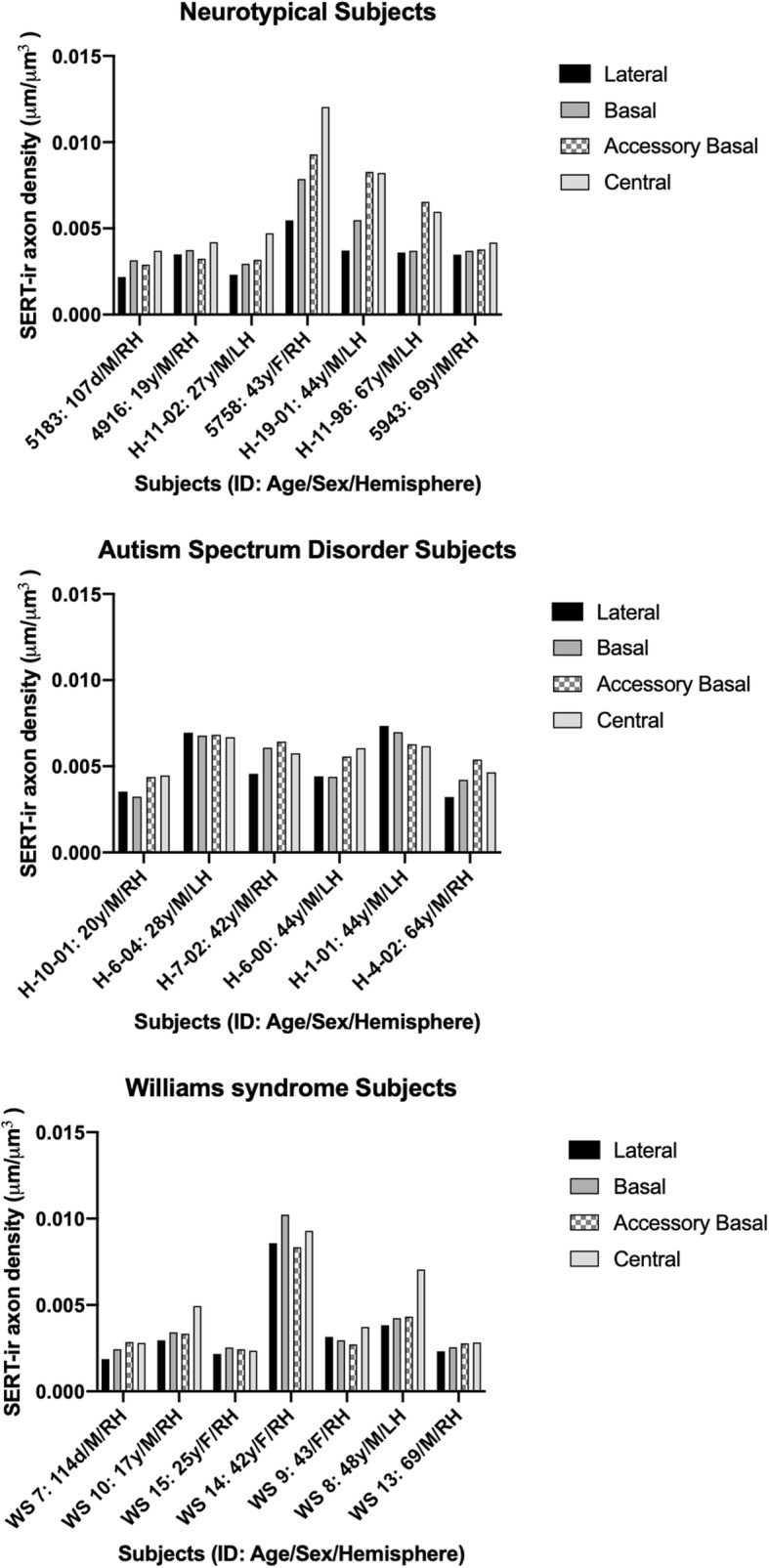
Stereological estimates of SERT-ir axon density in the lateral, basal, accessory basal, and central nuclei of the amygdala of individual subjects in each diagnostic group

**Fig. 3:**
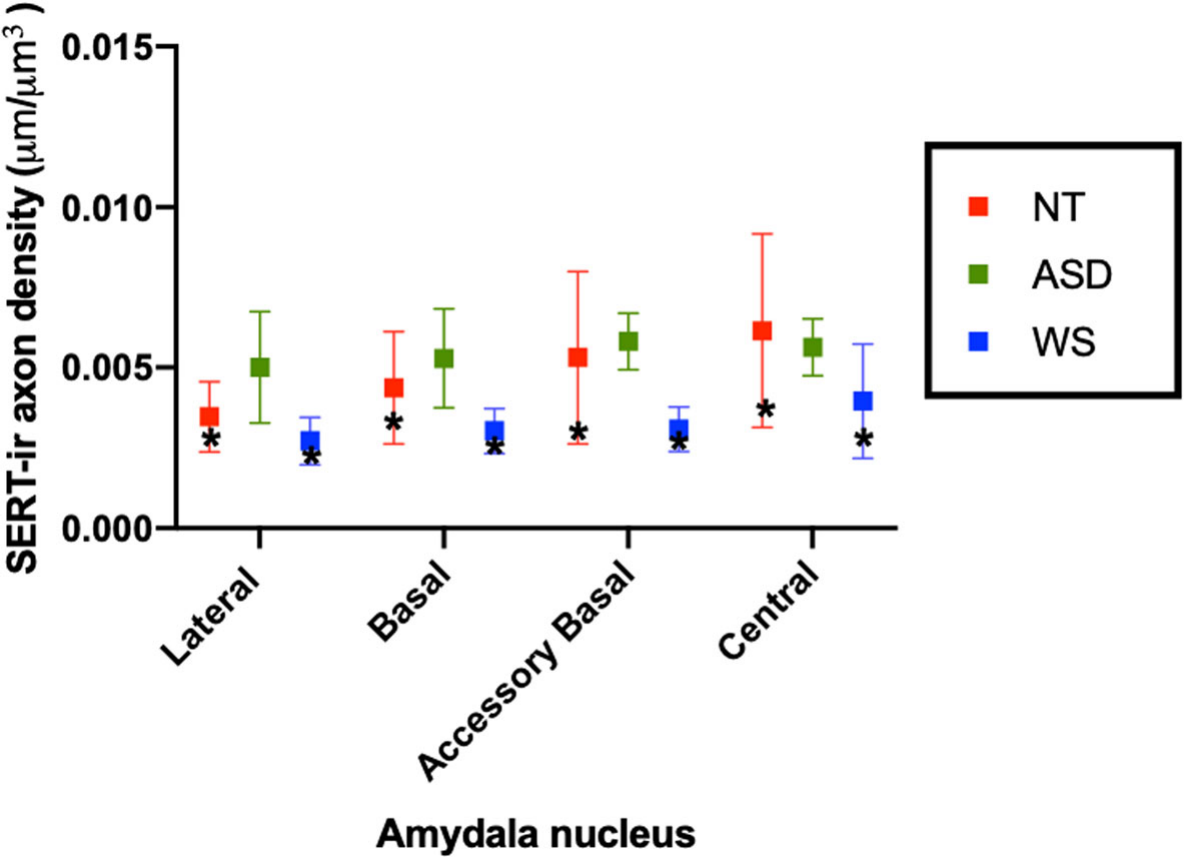
Mean SERT-ir axon density in the lateral, basal, accessory basal, and central nuclei of the amygdala in the adult subjects of each diagnostic group. The WS and NT adult means are overlaid by values of the WS and NT infant subjects (WS 7 and 5183, respectively) denoted by asterisks. Lines represent standard deviation of the mean. As observed in the figure, while WS and NT infant values are in the lower range of the adult values, they fall within the standard deviation of the adult mean

Stereological results of mean SERT-ir axon density and standard deviation in each nucleus in NT, ASD, and WS are reported in Table 2. One subject in the WS data set, WS 14, was found to be an outlier by the Grubbs outlier test and so was excluded from the WS mean values and all statistical analyses, although individual values of this subject are included in Fig. 2. No correlations were found between age, sex, or postmortem interval and SERT-ir axon density. As observed in our previous analyses of the postmortem amygdala in WS and ASD [7, 8], no significant differences in planimetric volume of any nucleus examined were found between the three groups. SERT-ir axon densities in the WS and NT infant subjects, although lower than the adult means, were within the standard deviation of the adults in their diagnostic group (Tables 2 and 3; Figs. 2 and 3).

**Table 2:** Mean SERT-ir axon density and standard deviation in micrometers (μm/μm^3^) in each nucleus of the amygdala in neurotypical, autism spectrum disorder, and Williams syndrome brains

Diagnosis	Mean SERT-ir axon density and standard deviation (μm/μm^3^)			
	Lateral	Basal	Accessory basal	Central
Neurotypical (adults only)	0.00368 ± 0.00101	0.00458 ± 0.00182	0.00572 ± 0.00270	0.00656 ± 0.00309
Neurotypical (all subjects)	0.00347 ± 0.0011	0.00437 ± 0.00175	0.00532 ± 0.00268	0.00615 ± 0.00302
Autism spectrum disorder	0.00501 ± 0.00174	0.00528 ± 0.00154	0.00581 ± 0.00088	0.00563 ± 0.00088
Williams syndrome (adults only)	0.00384 ± 0.00240	0.00432 ± 0.00296	0.00399 ± 0.00223	0.00504 ± 0.00268
Williams syndrome (all subjects)	0.00289 ± 0.00067	0.00314 ± 0.00071	0.00312 ± 0.00075	0.00418 ± 0.00188

**Table 3:** *P* values of NT compared to ASD, WS, and WS. Comparisons with ASD includes only the adult subjects in NT and WS due to lack of an infant age match in the ASD data set

Comparison	Lateral	Basal	Accessory basal	Central
NT vs ASD	0.4684	0.8470	> 0.9999	> 0.9999
NT vs WS	0.4200	0.1950	0.0513	0.3460
ASD vs WS	0.0425*	0.0466*	0.0365*	0.4693

* indicicates statistical significance *p* < 0.05

Mean SERT-ir axon density in ASD was greater than WS in all nuclei examined (Table 2; Fig. 3). The difference between ASD and WS reached significance in the lateral, basal, and accessory basal nuclei (*p* = 0.0425, *p* = 0.0466, *p* = 0.0365, respectively; Table 3). No significant differences were found between NT and ASD or NT and WS in any nucleus of the amygdala examined. In ASD, mean SERT-ir axon density was slightly increased in the lateral and basal nuclei, similar in the accessory basal nucleus, and slightly decreased in the central nucleus compared to NT (Fig. 3; Tables 2 and 4). Mean SERT-ir axon density was decreased in WS compared to NT in all four nuclei, and the difference between the two groups was largest and approaching statistical significance in the basal (*p* = 0.0513) and accessory basal nuclei (*p* = 0.0513; Tables 2, 3, and 4).

**Table 4:** % difference of mean SERT-ir axon density in ASD and WS compared to NT in each nucleus of the amygdala

Nucleus	ASD (%)	WS (%)
Lateral	+ 36.1	− 21.5
Basal	+ 15.3	− 31.4
Accessory basal	+ 1.6	− 45.5
Central	− 14.2	− 36.3

## Discussion

This is the first quantitative stereological study to examine serotonergic innervation of the major amygdala subdivisions in two neurodevelopmental disorders characterized by dichotomous socio-affective behavioral phenotypes. We found significant differences in serotonergic innervation of the amygdala between WS and ASD. Furthermore, WS and ASD displayed quantitative changes in opposing directions compared to neurotypical controls. These findings contribute to a growing body of literature [7–9, 18] in WS and ASD which demonstrate that both disorders display selective vulnerability of similar targets in the social brain, but in quantitatively opposing directions of change compared to healthy controls. This pattern parallels the dichotomous sociobehavioral phenotypes of the two disorders, suggesting that the microanatomical changes in neural structure of these regions may contribute to behavioral differences.

Specifically, in the present study, we found trends of a slight increase in mean SERT-ir axon density in ASD compared to NT and a decrease in mean SERT-ir axon density in WS compared to NT (Tables 2, 3, and 4; Fig. 3). Differences between WS and NT are greater than differences between ASD and NT in most nuclei, and the decrease in mean SERT-ir axon density in WS compared to NT approaches statistical significance in the basal and accessory basal nuclei (*p* = 0.0513 for both nuclei). Differences between ASD and WS are more robust: mean SERT-ir axon density in ASD is greater than WS in all nuclei examined, and as we predicted, these differences are significant in the basolateral nuclei, which demonstrate significant connectivity to association cortex, including the prefrontal cortex, another region preferentially targeted in both disorders [8, 41]. Additionally, the present dichotomous findings in the basolateral nuclei of the amygdala in WS and ASD parallel the dichotomy of change in neuron number in the same regions of interest in the two disorders: neuron number in the basolateral nuclei is decreased in ASD compared to NT [7, 18] and increased in WS compared to NT [9]. Given the role of serotonin in the regulation of several neurodevelopmental processes, including neurogenesis, neuronal differentiation, neuropil formation, axon myelination, and synaptogenesis [19, 42–45], perhaps the dichotomous amygdala pathologies observed in WS and ASD in these two domains, neuron number and SERT axon density, could be related to the effect of different manifestations of serotonergic disruption to amygdala cellular development and seemingly opposing behavioral phenotypes [46].

While the similar pattern of differences in behavioral phenotype and SERT-ir axon density in WS and ASD are intriguing, the relationship between serotonergic innervation of the amygdala and behavior is unclear. A recent study found that mice with homozygous and hemizygous knockouts of the SERT gene have increased anxiety, enhanced fear acquisition, and disrupted inhibition in the amygdala [29], indicating that a possible role of SERT in social behavior may be related to the modulation of reactivity of the amygdala in response to stimuli with emotional valence. Typical activation and reactivity of the amygdala in response to emotional stimuli, such as faces, is crucial to determining the emotional valence of stimuli for appropriate behavioral response. In humans, activation of the amygdala in response to faces in general, as well as positive emotion faces (such as “happy”) and negative emotion faces (such as “angry” or “fearful”), are parts of different emotional valence cascades which contribute to appropriate sociobehavioral response. Both individuals with ASD and individuals with WS have demonstrated atypical activation of the amygdala in response to human faces. Specifically, individuals with ASD display hyperactivation of the amygdala in response to human faces and are avoidant of the eye region, in which much of emotionally relevant social cues are displayed in humans, suggesting a negatively valenced overarousal of the amygdala in response to social stimuli in ASD which may contribute to social avoidance behaviors [5, 47–49]. Individuals with WS, in contrast, display hypoactivation of the amygdala in response to negative emotion faces and hyperactivation in response to positive emotion faces, suggesting disruption of autonomic processing in response to both positive and negative emotionally valenced stimuli, which may contribute to the atypically strong prosocial drive characteristic of the disorder [10, 50, 51].

Comparative studies examining serotonergic innervation of the amygdala in closely related species may help shed light on how different patterns of serotonergic innervation might contribute to socio-affective behavior. Bonobos and chimpanzees are two closely related apes and are the closest living relatives to humans. Bonobos typically respond to conflict with prosocial strategies [52, 53], while chimpanzees more frequently respond to conflict with aggression [54]. These behavioral differences are thought to be mediated in part by differences in emotional reactivity between species [55]. SERT axon density in the postmortem amygdala is lower in chimpanzees relative to bonobos and humans and more similar between bonobos and humans than between bonobos and chimpanzees [33, 40]. While species-specific differences are not directly comparable to differences across human neuropathologies, the observation that SERT-ir axon density in the amygdala is more similar in humans and bonobos, two highly prosocial species, than in phylogenetically close chimpanzees and bonobos, implicates the role of serotonergic innervation of the amygdala in social behavior more generally.

While the association between differences in serotonergic innervation of the amygdala and behavioral phenotype in WS and ASD are speculative, genetic evidence suggests disruptions to the serotonergic system are a feature of both disorders. One possible genetic link to serotonergic disruption of the amygdala is GTF2IRD1, a general transcription factor included in the WS deletion that is linked to the characteristic WS behavioral phenotype [56] and also implicated as a common site of allelic variation in autism [57]. Genetically altered mice with a deletion of GTF2IRD1 demonstrate altered serotonergic metabolism in the amygdala and frontal cortex, as well as reduced fear and aggression compared to wild-type mice [24, 26]. Another possible mechanism could be related to genetic variation of serotonin transporter genes, which have been linked to cognitive and behavioral differences in primates [58]. In addition to GTF2IRD1, several other genetic polymorphisms linked to ASD are found in genes involved in serotonin transporter signaling and function [59–62], and high concentration of serotonin in the blood, called hyperserotonemia, occurs in about one third of autism cases [63]. Blood levels of serotonin are normal in most cases of WS [64]; however, two separate studies have reported on a total of four cases in which patients with the common WS genetic deletion display hyperserotonemia, along with social and communicative deficits diagnostic of autism rather than WS [65, 66]. The researchers of the later study [66] also genotyped the SERT polymorphism (5-HTTLPR) for the two subjects they examined and found both were homozygous for the short allele (5-HTTLPR *s*). Tordjman and colleagues suggest that that the deviation from typical WS phenotype displayed by the two subjects in their study could be due to an interaction of WS genetic deletion with other genetic factors, such as the 5-HTTLPR polymorphism. The 5-HTTLPR polymorphism has been linked to socio-affective behavioral variation in humans and non-human primates [67, 68], and the 5-HTTLPR *s* allele is associated with heightened amygdala reactivity [69, 70] and stronger amygdala-prefrontal functional connectivity [71] in healthy subjects. Furthermore, the 5-HTTLPR *s* allele is thought to be a genetic risk factor for neuropathologies associated with deficits in affect and social behaviors [58, 72]. This polymorphism may contribute to the present findings in the WS and ASD amygdala, as well as the characteristic behavioral phenotypes. A future project aimed at genotyping the 5-HTTLPR polymorphism in the subjects of this study would shed light on the possible effects of this polymorphism to the WS and ASD phenotypes.

## Limitations

The sample size of this study is limited by the availability of tissue. The available material is further subjected to an elaborate immunochemical staining that often requires further exclusion of subjects to ensure that only highest quality tissue is used for data collection.

Despite this limitation, we found robust differences between the WS and ASD groups, suggesting differences between NT and the two disorders could potentially reach significance with the addition of a few more subjects.

## Conclusions

The present study is the first quantitative stereological study to examine serotonergic innervation of the major amygdala nuclei in two closely linked neurodevelopmental disorders with dichotomous atypical sociobehavioral phenotypes. We found that quantitative differences in SERT-ir axon density in the amygdala in WS and ASD parallel the opposing differences between the two disorders that we previously observed in neuronal distribution of the amygdala. Additionally, these dichotomous findings of atypical microstructure of the amygdala in WS and ASD parallel the dichotomous sociobehavioral phenotype of these two disorders. The serotonergic system is crucial to both neuronal development and behavioral modulation. The present findings of opposing disruptions to the serotonergic system in ASD and WS may contribute to differential atypical development of the amygdala and subsequent differences in amygdala reactivity to social stimuli in WS and ASD. Given the frequent use of SSRIs in patients with WS and ASD [22, 23], yet the relative lack of knowledge of the mechanisms involved, more studies that examine the role of serotonin in the etiology and phenotype of WS and ASD are needed to inform treatment and identify targets of future, more effective therapeutics in these disorders.

## Data Availability

The datasets used and/or analyzed during the current study are available from the corresponding author on reasonable request.

## Abbreviations

ASD: Autism spectrum disorder
NT: Neurotypical
SERT: Serotonin transporter
SERT-ir: Serotonin transporter immunoreactive
WS: Williams syndrome
